# A culturally adapted, social support-based, diabetes group visit model for Bangladeshi adults in the USA: a feasibility study

**DOI:** 10.1186/s40814-022-00974-9

**Published:** 2022-01-24

**Authors:** Megha K. Shah, Sukyi Naing, Nithin Kurra, Mary Beth Weber, Nadia Islam, Mohammed K. Ali, K. M. Venkat Narayan

**Affiliations:** 1 Department of Family and Preventive Medicine, Emory University School of Medicine, 4500 N. Shallowford Rd. Dunwoody, Atlanta, GA 30338 USA; 2 Hubert Department of Global Health, Emory University Rollins School of Public Health, Atlanta, USA; 3 Department of Epidemiology, Emory University Rollins School of Public Health, Atlanta, USA; 4 Division of Population Health, New York University Grossman School of Medicine, New York City, USA

**Keywords:** Asian Americans, Community-based participatory research, Diabetes mellitus, Type 2, Pre-diabetic state, Shared medical appointment, Health programs

## Abstract

**Background:**

Interventions focused on weight loss can prevent, delay, and improve management of type 2 diabetes (T2D). However, implementation of these programs is challenging in diverse populations. South Asians have higher risk for T2D, yet to date, there have been limited programs for this community in the USA. The aim of this project was to develop and test the feasibility of a tailored group visit model for Bangladeshis with type 2 diabetes (T2D) or prediabetes based in primary care.

**Design:**

Mixed-methods single-arm feasibility study.

**Setting:**

An academic health center-based primary care clinic in Atlanta, Georgia.

**Participants:**

Bangladeshi adults > 18 years old with T2D or prediabetes

**Methods:**

In conjunction with a community-academic board, we conducted focus groups to tailor an existing evidence-based curriculum to a culturally acceptable intervention. Fourteen participants enrolled in the 16-week program focusing on healthy diet, exercise, and weight loss. The primary feasibility outcomes were number of sessions attended and participant satisfaction with the intervention. Weight, blood pressure, cholesterol, and HbA1C were measured at beginning and end of study. Participants were asked to evaluate each session on level of satisfaction. One tailed paired *t* tests were used to test significance of pre-post changes in outcomes.

**Results:**

Key themes from the formative focus groups (*n* = 50) were closely tied to sociocultural beliefs and included: dietary patterns, physical activity perceptions, and healthcare access concerns. In the intervention, 10 of 14 participants had baseline and follow-up data. Participant attendance averaged 50%. Statistically significant reductions in mean weight (− 2%, 95%CI: − 3.1, 0.2 kg), systolic/diastolic blood pressure (− 12.7 mmHg [95%CI: − 23.2, − 2.2]/− 3.7 mmHg [95%CI: − 7.6, − 0.1], respectively), and triglycerides (− 62.6 mg/dl, 95%CI: − 123.1, − 2.0) were noted. Overall, participants reported high levels of satisfaction with the program.

**Conclusion:**

A lifestyle program based in primary care is feasible and acceptable for Bangladeshi immigrants. Larger studies testing the effectiveness of group programs, in primary care, to improve cardiometabolic factors are important.

**Trials registration:**

ClinicalTrials.gov; NCT03861546. Registered 28 February 2019.

**Supplementary Information:**

The online version contains supplementary material available at 10.1186/s40814-022-00974-9.

## Key Messages regarding feasibility

1. What uncertainties exist regarding feasibility?

- We wanted to explore how to adapt the National Diabetes Prevention Program for South Asians in the USA.
- We then wanted to investigate recruitment, retention, acceptability, and objective measures changes in weight and other cardiometabolic risk factors among South Asians with Type 2 diabetes or prediabetes to a group visit program adapted from the National Diabetes Prevention Program.

2. What are the key findings?

- The group visit model is feasible to deliver evidence-based diabetes prevention education when tailored for South Asians, who are at increased risk of diabetes, may improve acceptability.
- Retention was lower than expected however the intervention was highly acceptable.

3. What are the implications of the findings for the design of the main study?

- Incorporating a support partner in a group visit is feasible and highly acceptable for South Asian participants, and future interventions should explore how to improve retentions.

## Background

Evidence suggests that lifestyle changes that promote weight loss can improve glycemic control among adults with type 2 diabetes (T2D) and reduce incidence of T2D among those at risk [1–3]. Yet, the reach of such programs for adults at-risk has been low [4, 5]. Due to challenges with cultural adaptation, poor reach is especially pertinent for immigrants to the USA who are both at higher risk of T2D [6] and may not be culturally aligned with mainstream approaches for lifestyle modification.

South Asians are disproportionately affected by T2D compared to whites and other people of color [7, 8]. Bangladeshi are one of the fastest growing subgroups of South Asians in the USA [9] and face significant challenges related to English proficiency, cultural beliefs and practices, and socioeconomic status that may impact achievement of T2D prevention and care goals. For example, poor adherence to health provider recommendations related to language barriers and health literacy lead to a reduced likelihood of South Asian patients receiving guideline-based standards of care for T2D management [10]. Further, there is a lack of culturally tailored lifestyle programs and resources for Bangladeshi adults [11].

Primary care clinics may be the ideal hub to provide lifestyle education. Group-based clinic visits are a sustainable strategy to increase patient access by seeing more patients in a shorter time frame and increasing efficiency of the time clinicians spend with patients. In this model, individuals participate in a group visit with a portion of the visit dedicated to personalized care. The visits generally involves 10 to 16 patients in a 1- to 2-h visit in which a portion is facilitated by a team (e.g., a physician/nurse/nutritionist) in a group setting and a portion in which patients meet separately with a clinician [12]. The rationale for the group format is that it provides support that improves patient activation, thus group visits focus on educational interventions such as self-management, medication management, or nutrition. The group visit model has been shown to be feasible in several chronic health conditions, including T2D self-care and are associated with lower direct medical costs and higher guideline adherence [13]. Furthermore, these visits are reimbursed by all payers, creating a sustainable approach.

Given the need for cultural alignment and the disproportionate burden of T2D in Bangladeshi immigrants, and limited studies on group visits for South Asians in the USA, the purpose of this study was to (1) collect formative qualitative data through focus groups; (2) use the formative data to inform and culturally adapt an evidence-based program for diabetes self-management and prevention; (3) and implement the program to explore the feasibility and impact of a culturally-tailored, community-informed, group visit model in primary care to improve health behaviors among Bangladeshis with T2D and prediabetes in Atlanta, Georgia.

## Methods

### Study design

#### Guiding framework

The study used a community-based participatory research (CBPR) approach, in which stakeholders directly affected by the health condition with knowledge of the local context are equitable partners in the research process [14]. Our community advisory board (CAB), the Atlanta South Asian Health Alliance (ASHA), consisted of 14 academic and community partners representing the Atlanta Bangladeshi community. Members included religious leaders, representation from cultural organizations, community members with diabetes and prediabetes, clinicians in the community, and public health researchers. Specific CAB activities included development of research questions, participant recruitment, interpretation of qualitative data, and guidance on cultural adaptation of the lifestyle education program, which was based on the National Diabetes Prevention Program (DPP) [15].

#### Formative study phase

We conducted focus group discussions (FGDs), from October 2018 to January of 2019, to discern factors influencing lifestyle behaviors among Bangladeshis with T2D and prediabetes. The group environment enabled identification of community norms and socio-cultural behaviors [16].

Trained, gender-matched moderators of South Asian descent used a semi-structured discussion guide to assess views on influences of eating habits, engagement in physical activity, perceptions of the role of primary care, and insights on health programs. A notetaker was present at each FGD. Eight FGDs were conducted with two modes of stratification: age (18–39, ≥ 40) and sex [1 male and 1 female in the 18–39 category and 3 males and 3 females in the ≥ 40 category). Sex and age homogeneity was used to minimize within group hierarchies and foster open discussions [17]. We over-selected for those over 40 as those adults had a higher risk of diabetes [18]. Participant recruitment was led by a community coordinator with close ties to the community; recruitment methods included flyers, social media messaging, word of mouth, and engaging community leaders. Each group consisted of an average of 7 participants and lasted 60–90 min. Data from one female focus group was not used because of low participation. We conducted FGDs in restaurants and a local mosque. Before each FGD, we obtained written consent and assured confidentiality. At the conclusion of each FGD, participants completed surveys where they reported age, chronic health conditions, marital status, and education level. Participants received $20 gift cards to local supermarkets and refreshments.

#### Intervention adaptation phase

The intervention consisted of an adaptation of the DPP curriculum that involves lifestyle modification support to lower T2D risk [4]. Based on the FGDs and feedback from ASHA, researchers modified the curriculum. ASHA members and academic partners divided the DPP curriculum into 8 main areas, and small teams and met weekly to adapt each lesson. Structure and logistics of the group visit were decided by the entire team and reviewed monthly. Adaptations are described in the “Results” section and Supplemental Table 1.

#### Intervention program eligibility, recruitment, and setting

The feasibility study was a one arm, pre–post-trial with data collection at baseline and 16 weeks. The recruitment target was between 10 and 14 participants, which is considered the ideal size for a group visit model [19]. Individuals were eligible to participate if they (1) self-identified as Bangladeshi; (2) had self-reported prediabetes or T2D; (3) were between the ages of 18 and 75; (4) and could bring a partner with self-reported prediabetes or T2D to all in-person sessions. Participants were recruited from a single, academic, family medicine clinic, through physician referrals, and electronic health record data. Telephone screening was used to determine eligibility. The intervention lasted 16 weeks, with 8 in-person sessions. Check-ins via text message or calls were conducted during weeks when the group did not meet.

The intervention was delivered by a family medicine physician with training in lifestyle medicine and a Bangladeshi health coach. Each visit consisted of a group-based lesson and individualized care plan with the clinician to tailor goals for each participant. The in-person group sessions were 90 min. The setting was an urban academic family medicine clinic in Atlanta, Georgia. The family medicine center includes 10 full time faculty and 30 resident physicians with approximately 25,000 unique patient visits per year. All group visits took place in a classroom located in the clinic that was large enough to permit a circular seating arrangement for the patients, clinician, and coach. The protocol was approved by the Emory IRB in October of 2018. Recruitment for the qualitative study began in October 2018 and for the feasibility study in February 2019. All procedures were in accordance with the ethical standards of the responsible committee on human experimentation (institutional and national) and with the Helsinki Declaration of 1975, as revised in 2000. Informed consent was obtained from all participants included in the study.

#### Intervention outcomes

The primary feasibility outcomes of the intervention were number of sessions attended and overall satisfaction with the intervention. Secondary outcomes included weight change, as previous trials demonstrated that a modest amount of weight loss (~ 5–7%) can reduce the risk of progression to diabetes for adults with prediabetes and improve glycemic control among those with diabetes [1, 2]. Blood pressure, fasting lipids, and A1c were also included. Participants were encouraged to track the number of days they engaged in moderate physical activity.

### Analysis

#### Formative phase analysis

FGDs were audio-recorded, de-identified, and transcribed verbatim. All coding, organization, and data management were done in MaxQDA. We developed codes deductively and inductively and used an iterative team-based analysis process to create the codebook [20]. Three core reviewers reviewed all the transcripts, reviewers included 1 member from our CAB and 2 research team members (MKS, SN). All three reviewers created early memos of two high-quality transcripts. The core reviewing group reviewed the early memos and developed an initial codebook. The codebook was then validated on the same two transcripts, and the core reviewing group then met to refine the codes. Once the coding was consistent among reviewers, the refined codebook was applied to the remaining transcripts. The core reviewing group met weekly to discuss any coding issues; discrepancies in coding of > 10% were resolved through team consensus. Data saturation was reached by the 2nd focus group for each gender. The theory development phase was informed by the data collected and previous evidence [11, 21].

#### Intervention analysis

Quantitative analysis was conducted in Microsoft Excel and SAS, version 9.3 (SAS Institute, Cary, NC). Descriptive analyses were conducted for all variables and baseline and 16-week values were compared using paired *t* tests (for continuous variables). Adherence was measured by number of sessions attended. Given the small sample size, more advanced statistical testing was not appropriate.

## Results

### Formative phase results

Focus group participants (*n* = 50) consisted of 46% female (*n* = 23) and 54% male (*n* = 27). FGDs consisted of participants and research team members only. Most were married (> 75%) and had at least some college education (Table 1).

**Table 1:** Demographic characteristics of focus group participants by gender

Characteristic	Women *n* = 23 , *n*(%)	Men *n* = 27, *n*(%)
**Age, years**		
18–40	7 (30%)	8 (30%)
41–60	10 (44%)	15 (56%)
61–70	2 (9%)	3 (11%)
Missing	4 (17%)	1 (3%)
Mean age	42.1 years	47.8 years
**Marital status**		
Single	2 (9%)	3 (11%)
Married	18 (78%)	24 (89%)
Missing	3 (13%)	0
**Education level**		
High school degree	3 (13%)	1 (4%)
Some college	7 (30%)	2 (14.5%)
Bachelor’s degree	8 (35%)	12 (44%)
Master’s degree	3 (13%)	10 (37%)
Doctorate-level	2 (9%)	2 (14.5%)

### Influences of lifestyle behaviors

Three major categories of lifestyle influences were prevalent. All were closely tied to sociocultural beliefs: dietary patterns, physical activity perceptions, healthcare access.

### Dietary patterns

Dietary schedules were a noted barrier, “(...) we eat dinner so late. And lunch a little early. What happens is that you end up eating a lot of snacks in between, which you might not had you had dinner a little earlier, so you end up eating a lot more than you actually need” (≥ 40 male).

The relationship between dietary behaviors and sociocultural practices includes high consumption of deep-fried foods, refined carbohydrates, and excessive oil in cooking. Participants described these practices as difficult to change. A male participant (≥ 40) stated, “We cannot live without rice. That is the problem. I tried many times (...) but we stopped many times."

Social gatherings (i.e., “parties”), are an important means of community-building and maintenance. Unhealthy dietary behaviors are reinforced during parties with high intake of refined carbohydrates, sugar, and oily foods. This includes calorie-rich evening snacks with tea in social gatherings and at home. Despite awareness of unhealthy dietary patterns, participants indicated feelings of discomfort changing behaviors associated with traditional norms, especially in social settings.

Wives and mothers often decide meals. Some females (≥ 40) noted that by cooking “to the man’s liking”, males also influence dietary decisions. Participants (≥ 40) disclosed that US-born children influence parents’ dietary behaviors, specifically because they usually prefer dining out and fast food.

### Perceptions of physical activity

The females engage in less exercise than their male counterparts. A few participants (≥ 40) noted that females in the Bangladeshi community do not exercise. Many also cited lack of motivation as a barrier to physical activity. Specifically, within the younger male (< 40) group, those who do not exercise regularly noted laziness, jobs, and family life as barriers.

Male and female participants communicated differing perspectives on physical activity. A female participant (< 40) noted mixed-gender environments as a barrier to exercise, “(...) in our culture, we don't want to be men and women together at the gym (...) that's probably why a lot of women in our culture don't work out … ”. Females described engaging in physical activity with family members and friends as motivational. There was a shared belief among males and females that role models helped to motivate participation in exercise. A male participant (≥ 40) stated, “So we have to create in our community, some sort of models or leaders who can take us or take in this regard to take care of [our] health exercise issues."

Participants shared a widespread belief that Islamic prayer is good form of exercise; many cited that this was enough to meet daily exercise requirements, “Talking of the imam, he always tells us do the five times Salat, that is very good exercise” (≥ 40 male).

### Healthcare access

Participants commonly stated insurance costs as a barrier to healthcare access. Those who are affected by negative health outcomes and inability to afford health insurance: “So what they are supposed to do when people have brain strokes, blood pressure [and blood sugar are] high ( … ). [He doesn’t get treatment] because he doesn’t have insurance” (≥ 40 male). This is compounded by limited knowledge of how to navigate the healthcare system, “And being immigrant ( … ) many of us still don't understand the system. How it works" (≥ 40 male). Participants also communicated limited knowledge about insurance options. Participants cited physicians as trusted sources of information, noting that community members often seek medical advice from family members or friends who are health professionals.

### Recommendations for culturally tailored health programs

Some participants doubted community members would attend health programs without adequate incentives, including individualized attention from the physician. Other facilitators included easy access to health information, easy enrollment processes, scheduling that accounts for participants’ jobs, and clinicians or translators from their community. Participants emphasized cultural relevancy as a critical factor when developing health programs and cited weekly meetings as a barrier to attendance.

#### Intervention adaptation results

Based on formative data from FGDs in the community, adaptations were made to the DPP curriculum. All 16 core content sessions were maintained but condensed into 8 in-person sessions and culturally adapted to align with practices of the Bangladeshi community [15]. For example, sessions focused on mindfulness included Islamic teachings, incorporated by guidance from ASHA. Sessions on exercise focused on home exercise programs. For females, the education focused on how to increase aerobic activity while doing house chores. Diet recommendations were modified to focus on schedules and portion control rather than diet substitutions, adjustments to native dishes, and management of diet during Islamic holidays. Format adaptations included requiring participants to bring a support partner to enhance social support and reducing in-person sessions to every other week (8 in person sessions, 8 phone check-in sessions). All sessions were in English, and an interpreter was available at each in-person session to assist when needed. See Supplemental Table 1 for a full list of adaptations.

#### Intervention trial results

**Fig. 1:**
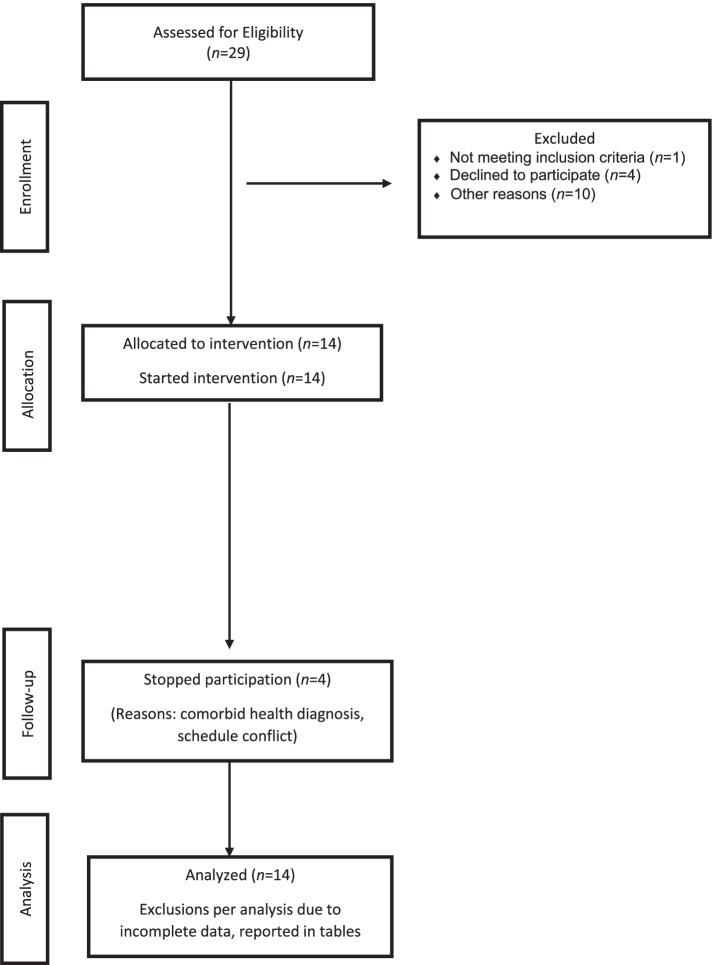
Modified CONSORT flow diagram for DoST Intervention

Figure 1 demonstrates eligibility and enrollment of participants. Twenty-nine adults were identified as eligible, based on physician referrals and electronic health record data. When contacted, one was reported not having diabetes or prediabetes, and 4 declined, giving no reason for declining. Ten declined for other reasons including transportation barriers (4), work conflict with the time of the group sessions (3), and out of the country during the study period (3). Seven dyads, (3 male/female married couples, 1 father/son, 1 mother/daughter, 2 same gender peers) consented and enrolled in the feasibility study, DoST (Diabetes Stops Together) (clinical trials: NCT03861546). Two dyads, one married couple and 1 same gender peer dyad, dropped out of the study during the intervention period, one reported health reasons, and another reported a change in schedule thus, could no longer attend in-person sessions. Among the dyads that dropped out, 2 had diabetes and 2 had prediabetes (see Fig. 1 and Table 2).

**Table 2:** Demographic characteristics of the DoST pilot intervention study participants at baseline

Characteristics	Mean (SD) or % *n* = 14	Range
Gender (in % of total)		
Males	43%	–
Females	57%	
Age	50	23-74
Maximum education level (in %)		
Elementary school	8%	
Junior high school/some high school	0%	
High school or GED	23%	
Technical/vocational school/associates degree	0%	
Some college or university	8%	–
College or university graduate	46%	
Graduate level/advanced degree	8%	
No formal education/never attended school	8%	
HbA1C level in blood (in %)	6.3 (1.0)	5.1–8.9
Weight (kg)	75.3 (12.6)	57.45–99.18
Systolic blood pressure (in mmHg)	135 (23.7)	110–190
Diastolic blood pressure (in mmHg)	80.2 (8.4)	60–92
Body mass index (in kg/m^2^)	29.8 (4.9)	23.4–40.7
% reported previous history of:		
High blood pressure	54%	
High blood cholesterol	62%	
High blood sugar	77%	-
Dental problems	46%	
Breast cancer	0%	
Colon cancer	0%	

Key: *HbA1c* =hemoglobin A1c, *GED* = graduation equivalency diploma, *SD* standard deviation

Data collected in 2019 from an urban, academic, and family medicine clinic

Five dyads were included in the final analysis. Participants were 57% females, 43% were males, with a mean age of 50.1 (range: 23–74). Mean A1c was 6.26% (range: 5.1–8.9%) (Table 2). All participants had a diagnosis of prediabetes or T2D by self-report.

Eight of 10 participants attended at least 50% (range for all participants: 25–100%) of the in-person sessions. Mean weight change was -1.44 kg [-2.0%] (95%CI: − 3.1, 0.2)); with 8 of 10 participants losing weight by end of study. Participants’ pre-to-post mean systolic and diastolic blood pressures also decreased (systolic: − 13 mmHg (95%CI: − 23.2, − 2.2); diastolic: − 4 mmHg (95%CI − 7.6, − 0.1). There was no significant change in A1c. Triglycerides decreased by 62.6 mg/dL (95% CI: − 123.1, − 2.0) (see Table 3).

**Table 3:** Cardiometabolic risk factors from baseline to end of study

Variable	Baseline for participants with follow-up data	End of study	Change for participants with follow-up data^a^	95% CI
	(***n*** = 10)	(***n*** = 10)		
**Glycemic status**				
HbA1c (%)	6.1(0.6)	6.1(0.6)	− 0.04 (0.62)	− 0.5, 0.5
**Anthropometry**				
Weight (kg)	77.1 (13.6)	75.7 (13.0)	− 1.44 (2.29)	− 3.1, 0.2
% change			− 2.0%	
**Other cardiometabolic markers**				
Systolic blood pressure (mmHg)	134 (19.6)	121 (9.0)	− 13 (14.6)	− 23.2, − 2.2
Diastolic blood pressure (mmHg)	82 (6.3)	78 (7.5)	− 4 (5.0)	− 7.6, − 0.1
Total cholesterol (mg/dl)	169 (43.6)	162.3 (33.3)	− 6.7 (25.1)	− 24.7, 11.3
HDL (mg/dl)	45.3 (22.5)	45.8 (21.7)	1.0 (6.7)	− 4.1, 5.1
Triglycerides (mg/dl)	204.5 (84.8)	141.9 (62.8)	− 62.6 (84.7)	− 123.2, − 2.0

*n* (%) or mean (standard deviation)

*HbA1C* = hemoglobin A1C, *HDL* = high-density lipoprotein

Data collected in 2019 from an urban, academic, and family medicine clinic

^a^Represents mean of absolute change for participants with baseline and follow-up data

Participants self-reported an increase in number of days in which they engaged in moderate intensity exercise through self-journaling data. Overall participants reported high levels of satisfaction with the program and individual sessions in which a support partner was invited (consistently 5/5 on Likert-like scale for all sessions). No harms or adverse events were reported by participants.

## Discussion

This feasibility study, guided by CBPR, used mixed methods to plan, design, implement, and test the feasibility and acceptability of a primary care-based group visit for lifestyle management of prediabetes and T2D among Bangladeshis. Formative focus group discussions revealed the overlap of dietary patterns, physical activity, healthcare access, and sociocultural considerations. The intervention, based on previous evidence, showed feasibility to recruit Bangladeshi to participate in a group visit model. For those that completed the intervention, there were reductions in weight, blood pressure, and plasma lipids, similar to those seen in previous trials of lifestyle interventions [22, 23].

The formative qualitative results are consistent with previous work in South Asian communities, describing the impact of culture on behaviors, such as exercise habits, food preparation, and the importance of family responsibilities [24–28]. However, while previous studies identify a lack of knowledge of how lifestyle behaviors influence risk of T2D, participants described understanding how their cultural practices increased their risk of T2D. This study adds to the literature of South Asian diaspora by identifying novel barriers and facilitators to participant engagement. Qualitative work highlighted insurance as a major barrier to accessing care; this has not been reported in previous studies of South Asian immigrants [29]. Further, community members highlighted high levels of trust in physicians and preferred physician-led programs. This is in contrast to studies other ethnic minorities in which there are high levels of healthcare distrust, that may be associated with poor health outcomes [30].

The DoST feasibility study highlights the use of a clinic-based group visit model, as a potential model to engage vulnerable populations in lifestyle programs. Group visits have been shown to improve glycemic control in adults with T2D as well as improve T2D preventive care guideline adherence [13]. These findings have been replicated in low resource communities, however there is data lacking from Asian populations in the USA [31]. Our study demonstrates feasibility of the group visit for South Asian adults. The acceptability of a support partner in the group visit model is also consistent with previous literature that supports the use of group formats and spouses in DPP-like interventions [32]. However, to our knowledge, no interventions have tested the feasibility of embedding a support partner in a clinic-based group visit for a South Asian group.

In our experience, this study highlights some logistical considerations and novel findings. First, we were only able to offer the group visits one weekend day per month, while this worked for most, having other times or dates that the sessions were offered may have led to higher retention. Second, physical activity and dietary data were limited to qualitative self-report in our feasibility study, but more robust, objective measures would enhance the findings of the study and should be included in larger trial. Third, though the clinical outcomes are secondary outcomes, it was feasible to collect point of care finger prick testing at the first and last session of the intervention. Lastly, while previous studies of the diabetes prevention program demonstrate low male participation, 43% of the DoST participants were male, demonstrating higher male participation than other diabetes prevention programs [5, 33].

This study has several limitations. In the formative phase, we initially completed 8 focus groups, however, did not use data from one female group, that had only 3 participants. Our qualitative analysis revealed thematic saturation after the second female focus group, thus 3 female focus groups was assessed to be adequate. The focus groups were only conducted in English. The feasibility study was small, recruited from one clinic, did not include a comparison arm, and there was loss to follow-up, thus subject to bias. However, those that withdrew from the study reported conflicts with the date and time of the program and health reasons. Furthermore, 10 participants is within the ideal size for a group visit model [34]. Fourth, change in clinical outcomes should be interpreted with caution as, this study was not powered to detect a pre-specified effect size. However, these data will be used to power a larger effectiveness trial. The focus of this study was on first-generation Bangladeshis and may not apply to second-generation Bangladeshi-Americans.

## Conclusion

Although this feasibility study was small, it has promising results for the adaptation of lifestyle interventions, embedded in primary care. To our knowledge, this is the first cultural adaptation of a lifestyle program for South Asians, delivered within primary care using a group visit model that includes a clinician. By adapting a group visit model, the intervention has the added opportunity of sustainability, as these were reimbursable visits by all payers [35]. Also, by engaging our CAB in all aspects of adaptation, we improved acceptance for and engagement in the program. Lastly, participants reported high levels of satisfaction with the group visit format, inclusive of a partner to support their lifestyle change and engagement.

### Practice implications

Engaging adults in lifestyle change is challenging, especially among immigrant populations in which culture and language may create barriers. Utilizing resources within primary care to better meet the needs of specific populations may be an effective and sustainable strategy to reach certain populations. Our study demonstrates feasibility to engage South Asian adults in a lifestyle program embedded in primary care. Adaptations to existing evidence-based programs have the potential to improve the reach of lifestyle education and may improve uptake. For example, our study highlights feasibility to engage support partners, which may improve acceptability and reach to the targeted population [36, 37]. However, attention should be given to the resources needed to retain and accommodate participants to maximize session attendance, which may include offering multiple dates and times for group visits. Larger trials to assess implementation and effectiveness of group visits that utilize social support in primary care for lifestyle management among South Asians are needed and have broader application to other immigrant communities.

## Supplementary Information

**Additional file 1: Table S1.** Dietary behaviors.

## Data Availability

The datasets generated and analyzed during the current study are not publicly available due the small sample size and risk of breach of confidentiality but are available from the corresponding author on reasonable request.
