# Supplementary material for: A culturally adapted, social support-based, diabetes group visit model for Bangladeshi adults in the USA: a feasibility study
Source: Pilot Feasibility Stud. 2022 Jan 24;8:18. doi: 10.1186/s40814-022-00974-9 (PMC8785445; doi:10.1186/s40814-022-00974-9)
Supplement: Supplementary file 1 — Additional file 1: Table S1. Dietary behaviors. [file 40814_2022_974_MOESM1_ESM.docx]

**Supplemental Table 1**

| Theme | Example quotations | Adaptation considerations |
| --- | --- | --- |
| **DIETARY BEHAVIORS** | | |
| 1. Dietary schedule contributing to unhealthy behaviors | "Another thing I think is the fact that we eat dinner so late. And lunch a little early. What happens is that you end up eating a lot of snacks in between, which you might not had you had dinner a little earlier, so you end up eating a lot more than you actually need." [Male] | Emphasize consuming meals at routine times and scheduling of meals for optimal health and metabolic functions |
| 2. Rice consumption embedded in sociocultural context | "We cannot live without rice. That is the problem. I tried many times, "Okay, I'm not going to eat rice anymore," but we stopped many times. Could not continue enough. We had to go back. Sometimes we started like brown rice or quinoa or those kind of, but for the time being. Again, you go back because that is the thing you like most. The regular rice." [Male] | Prioritize portion control. Pair portioned rice intake with other nutrient-rich foods (lentils, vegetables, other grains, etc.) |
| 3. Women as primary dietary decision makers among older generation | "My mom cooks in our house, and because I'm out all day, it's kind of like my mom's helping me out and I don't have to worry about what I'm going to eat at the end of the day. So it's like when I come home, I'm just open to whatever is at the table." [Female]; "Because when my wife cook the food, if my children maybe don't like to eat. But we have to eat because she cooked so she influenced us too." [Male] | Engage women in the household as agents of diet change. |
| 4. Social gatherings ("parties") as contributing factor | "But I will say friends too. Because we have a habit of having a chicken party every weekend. Every weekend there is two or three chicken parties we will go to. And it is nothing but Bangladeshi food. Rice, [biriyani], eggs, lots of oily fish, oily, everything is oily." [Male]; | Incorporate tips about healthy eating when around friends |
| 5. First-generation children's preference of fast food and non-Bangladeshi food | "And the kids they don't like to eat in the house, okay. Mostly they just call and get the fast food you know, burger or chick-fil-a or something like that. They like those things. I like to eat at home... my wife also. But my kid, they say 'No, we don't like it just get some food from outside.' Because of my family, because of our kids, our eating habit changes." [Male] | Discuss health as a family and include all family members in lifestyle change |
|  |  |  |
| **EXERCISE** | | |
| 1. Women's perspectives on physical activity | "So one thing I think she's referring to is like in our culture, we don't want to be men and women together at the gym. So they want to go to women's separate only...So that's probably why a lot of women in our culture don't work out because they want to stay home or work out at home. They walk around their neighborhood." [Female] | Implement culturally sensitive programming: e.g., separated by gender, cultural music/ dance |
| 2. Physical activity among women | "Particularly from the ladies side, culturally in Bangladesh, the woman do not do exercise." [Male]; “Yeah, doing it with friends and family can help a lot. It will be like a game." [Female] | Emphasize household activities that are more likely to provide moderate aerobic activity. |
| 3. Men's perspectives on physical activity | "So we have to create in our community, some sort of models or leaders who can take us or take in this regard to take care of her health exercise issues." [Male] | Implement same-gender exercise/sports groups with leadership |
| 4. Physical activity among men | "So as a community we play volleyball. At least once a week, but I try to run like, at least three days a week. Play some soccer sometimes." [Male]; "I do walk a lot. Every day, I try to walk at least 45 minutes, at least." [Male] | Incorporate team sports into program/intervention. |
| 5. Perspective of namaz* as exercise | "Talking of the imam, he always tells us do the five times Salat, that is very good exercise." [Male]; | Involve religious leaders to educate and inspire the community |
| 6. Other barriers to physical activity | "I wanted to do some exercise, but I have no time to, because I'm super lazy, so I don't do any cardio or exercise. But before I mean I used to do some cardio, and would normally play once a week." [Male, <40] | Consult community members to discern which habits they want to implement to make healthy behaviors easier. |
| **HEALTHCARE ACCESS** |  |  |
| 1. High insurance cost as barrier | "Yes, I have health insurance, but my children doesn't have health insurance… so for my two children, I can't afford." [Female]; "So what they are supposed to do when people have brain strokes, sugar is high and blood pressure is high and all kinds of levels are not right…They don't do it because it was too expensive. Because he doesn't have insurance. Also you can apply for Medicaid, but it is pending." [Male] | Provide information on free or low cost programs in the community |
| 2. Limited knowledge about how to access care in the US | "And being immigrant, if you're talking about South Asian, many of us still don't understand the system. How it works." [Male] | Offer education on healthcare system in the US and state of residence |
| 3. Community members usually seek advice from physicians their friends/family know | "My family usually talks to someone we know that he is a doctor. He is in a specific field, but we prefer to talk with him, even if he is not in this field that we this problem." [Female] | Incorporate clinician and family members in the intervention |
| **HEALTH PROGRAMS** |  |  |
| 1. Facilitators | "Easy access. So there has to be doctors, if they get information easily, and process of enrollment is kind of easy, then it will be helpful to them. And also flexible time schedule to accommodate their work and everything." [Male]; "Like the language barrier, it would help if someone had a Bengali. Like for the elderly also, to have a Bengali doctor there, it helps a lot." [Female] | Ensure health programs: are culturally appropriate (including Bengali translator/Bangladeshi representatives), have a component of socialization, offer consultation opportunities with physician/specialist, are easy to access (date, time, location), are transparent about the type of program that is held, offer free medical testing for relevant measures, offer appropriate food options (e.g., healthy food options for the Bangladeshi palate, healthy snacks), include gift cards/small gifts as incentives |
| 2. Barriers | "What I'm saying is if you have an announcement or such and such doctor will have this health seminar, they will not show up. Like when they go for visit, that's the time you have a captive audience. That time but extra time you have to have enough incentive." [Male]; "So many families came and they just come to see any cultural program on there but instead of when they saw health program is going on they gonna at least laugh." [Male]; "...another thing is a lot of people, they don't even know whether they have diabetes or not." [Female] |  |

**Namaz*-Islamic prayer observed 5 times daily
